# *Staphylococcus aureus* Avoids Autophagy Clearance of Bovine Mammary Epithelial Cells by Impairing Lysosomal Function

**DOI:** 10.3389/fimmu.2020.00746

**Published:** 2020-05-05

**Authors:** Na Geng, Xiaozhou Wang, Xiaohui Yu, Run Wang, Yiran Zhu, Meihua Zhang, Jianzhu Liu, Yongxia Liu

**Affiliations:** ^1^College of Veterinary Medicine, Shandong Agricultural University, Tai’an, China; ^2^China Animal Health and Epidemiology Center, Qingdao, China; ^3^Research Center for Animal Disease Control Engineering, Shandong Agricultural University, Tai’an, China

**Keywords:** *Staphylococcus aureus*, bovine mammary epithelial cells, intracellular infection, autophagy, lysosomes

## Abstract

In dairy herds, mastitis caused by *Staphylococcus aureus* is difficult to completely cure on the account that *S. aureus* can invade bovine mammary epithelial cells (BMECs) and result in persistent infection in the mammary gland. Recent studies have demonstrated that autophagy can participate in cell homeostasis by eliminating intracellular microorganisms. The aim of the study was to investigate why *S. aureus* can evade autophagy clearance and survive in BMECs. The intracellular infection model was first constructed; then, the bacteria in autophagosome was detected by transmission electron microscopy. The autophagy flux induced by the *S. aureus* was also evaluated by immunoblot analysis and fluorescent labeling method for autophagy marker protein LC3. In addition, lysosomal alkalization and degradation ability were assessed using confocal microscopy. Results showed that, after infection, a double-layer membrane structure around the *S. aureus* was observed in BMECs, indicating that autophagy occurred. The change in autophagy marker protein and fluorescent labeling of autophagosome also confirmed autophagy. However, as time prolonged, the autophagy flux was markedly inhibited, leading to obvious autophagosome accumulation. At the same time, the lysosomal alkalization and degradation ability of BMECs were impaired. Collectively, these results indicated that *S. aureus* could escape autophagic degradation by inhibiting autophagy flux and damaging lysosomal function after invading BMECs.

## Introduction

Mastitis, a type of inflammation that occurs in the mammary parenchyma, can be induced by physical, microbial, and chemical factors, and it is a highly prevalent disease in dairy cows (1). Satisfactory evidence reveals that almost all cases of mastitis are caused by microorganisms (2). Infectious mastitis adversely affects milk quality and quantity and comprises a reservoir of microorganisms that spread the infection to other animals within the herd (3). The most common of such microorganisms is *Staphylococcus aureus* (4, 5). *S. aureus* mastitis possesses the characteristics of low cure rate and low pathogen elimination (6, 7). *S. aureus* can become walled off in the udder cell by thick, fibrous scar tissue so that the antibiotic cannot reach the bacteria. Even microbes that are sensitive to the antibiotics used may be unable to achieve the desired therapeutic effect (7).

Autophagy acts as a “cell guard” to clear intracellular pathogens involved in homeostasis (8–10). Autophagy occurs in the following three steps: formation of autophagosomes, then formation of autolysosomes by fusion between autophagosomes and lysosomes, and finally degradation of the cargo within the lysosomes (11). The complete autophagy flux starts from the autophagosomes that form the double-membrane structure. The key step for autophagy to produce biological effects is the formation of autolysosomes by fusion between autophagosomes and lysosomes (12). Lysosomes are monolayer-coated vesicles containing diverse acidic hydrolases (13), which can eventually degrade a variety of pathogens in autolysosomes.

Beclin1 does not only work with Atg14L to regulate the initiation of autophagy (14) but also combine with other proteins and form complexes to regulate the maturation and transport of autophagosomes (15, 16). Following the generation of LC3, the C-terminal fragment of LC3 is immediately cleaved by Atg4, a cysteine protease (17). The cleavage yields its cytosolic form LC3-I and exposes the carboxyl terminal Gly. LC3I is further activated by Atg7 (an E1-like enzyme), transferred to Atg3 (an E2-like enzyme), and finally modified into a membrane-bound form, LC3II (18). LC3II subsequently binds to autophagy vesicles and participates in autophagy activation. After binding with the polyubiquitinated proteins and LC3, SQSTM1/p62 performs the function of a junction protein to send the ubiquitinated protein into autophagy vesicles and degrade in autolysosome (19). Similarly, lysosomal-associated membrane protein 2 (LAMP2) is the main protein on the lysosome membrane. LAMP2 not only plays an important role in protecting the integrity of the lysosome membrane but also participates in regulating the fusion of autophagy vesicles and lysosomes (20). Cathepsins D (CTSD) and cathepsin L (CTSL) are essential components of functional lysosomes (21). The acidic environment in lysosomes plays an important role in maturating and activating lysosomal hydrolases and finally in degrading cargo in lysosomes.

Recent studies have reported that after *S. aureus* infection, autophagosomes are formed, but the autophagosomes and lysosomes cannot fuse normally to form autolysosomes, thus avoiding autophagy degradation (10, 22). The resistance of bovine mammary epithelial cells (BMECs) to *S. aureus* infection and induction of autophagy has not been thoroughly explored. Therefore, in this study, the characteristics of autophagy induced by *S. aureus*-infected BMECs was systematically described by detecting autophagic flux-related indicators and assessing lysosomal functions.

## Materials and Methods

### Reagents and Antibodies

4’,6-Diamidine-2’-phenylindole dihydrochloride (DAPI) and acridine orange (A8120) were purchased from Solarbio (Beijing, China). Bicinchoninic acid (BCA) protein assay kit and enhanced chemiluminescence (ECL) kit were obtained from Thermo Fisher Scientific Pierce (Rockford, IL, United States). LysoTracker Deep Red and Enhanced Cell Counting Kit-8 (CCK-8, C0042) were from Beyotime Biotechnology (Shanghai, China). Lipofectamine 2000 Transfection Reagent (L3000015) was purchased from Invitrogen (Rockford, IL, United States). Plasmid extraction kit was from TIANGEN Biotech (Beijing, China). Lysostaphin was from Sangon Biotech (Shanghai, China). Glutaraldehyde, formaldehyde, osmium tetroxide, and epoxy (low viscosity) resin were from Merck Millipore Company (Billerica, CA, United States).

The following primary antibodies were used: anti-p62/SQSTM1, anti-glyceraldehyde 3-phosphate dehydrogenase (GAPDH), and anti-CTSL/major excreted protein (MEP) were purchased from Abcam (Cambridge, MA, United States); anti-LC3B, anti-LAMP2, and anti-β-actin were obtained from Beyotime (Shanghai, China); anti-α-tubulin, anti-Beclin1, and anti-CTSD were from Proteintech (Chicago, IL, United States); Peroxidase-Conjugated AffiniPure Goat Antimouse IgG (ZSGB-BIO, Beijing, China); and goat antirabbit IgG (CWBIO, Beijing, China).

### Bacterial Strains and Cell Culture

*Staphylococcus aureus* strains (ATCC 25923) were cultured in Luria–Bertani (LB) broth at 37°C for 12 h. After reaching OD600 = 0.8–1.2, the bacteria were washed with phosphate-buffered saline (PBS) thrice to treat the cells. The bovine mammary epithelial cell line (MAC-T) was digested with trypsin at 37°C for 5 min and centrifuged at 1,000–2,000 r/min for 5 min. The MAC-T cells were then maintained overnight at 37°C in 5% CO_2_ without antibiotics in Dulbecco’s modified Eagle’s medium (DMEM), supplemented with 10% (*v*/*v*) heat-inactivated fetal bovine serum until the cell density reached 80%.

### Cell Viability Assay

MAC-T cells were seeded into 96-well plates (1 × 10^4^ cells/well) in 100 μl of DMEM medium. Twenty-four hours later, cells were infected with *S. aureus* [multiplicity of infection (MOI) = 8] for 2, 4, 6, and 8 h to assess the damage of the cells. The cell viability assay was performed using CCK-8 following the manufacturer’s instructions. The absorbance was read at 450 nm by the microplate reader (Sunrise, Salzburg, Austria).

### Immunofluorescence Staining

Cells were seeded on sterile coverslips placed in 24-well plates. The cells were then infected with *S. aureus* for 2 h, and they were fixed with 4% paraformaldehyde for 8 min, permeabilized with 0.2% Triton X-100 in PBS for 5 min, and blocked with 5% skimmed milk for 1 h at room temperature (RT). Slides were first stained with anti-α-tubulin antibody (1:200 diluted in PBS) for 2 h at RT. Cells were washed by PBS thrice and then incubated with peroxidase-conjugated AffiniPure (1:100 diluted in PBS) secondary antibody for 1 h and washed with PBS again. The nuclei were stained with 100 μl DAPI (blue) and washed thrice with PBS. Finally, all slides were mounted with ProLong Gold antifade mountant. Images were conducted on the Leica TCS SPE confocal microscope with a × 63 (1.3 numerical aperture) oil immersion objective. Images were taken at laser wavelengths of 555 and 488 nm. Images for colocalization analysis (percentage of protein–protein colocalization) were assessed using the JaCoP plugin in ImageJ after thresholding of individual frames. All colocalization calculations were performed on three independent experiments with 20 cells per condition in each experiment.

### Transfection

Ad-GFP-LC3B and Ad-mCherry-GFP-LC3B were obtained from Beyotime (Shanghai, China). MAC-T cells were prepared using Lipofectamine 2000 with 4 μg of DNA per well on a six-well plate transfected with Ad-GFP-LC3B and Ad-mCherry-GFP-LC3B when the cell density reached roughly 70% confluence. After transfected for 36 h, the cells were infected with *S. aureus* and observed with the confocal microscope (TCS SPE, Leica, Germany). Representative cells were selected and photographed. Twenty cells per condition from three independent experiments were applied for statistical analysis.

### Western Blotting

After infected with *S. aureus* for 2, 4, 6, and 8 h, MAC-T cells were collected and lysed in radioimmunoprecipitation assay (RIPA) buffer solution on ice for 30 min. After centrifugation at 12,000 × *g* for 15 min, the concentration of the protein samples was measured by BCA assay kit. Through sodium dodecyl sulfate (SDS)-polyacrylamide gel electrophoresis, the proteins were transferred to polyvinylidene fluoride (PVDF) membranes using a semidry blotting system. The blots were blocked in Tris–buffered saline (TBS) containing 5% skimmed milk powder. Membranes were incubated overnight at 4°C with the primary antibodies. Washed three times with Tris–buffered saline buffer with Tween 20 (TBST), membranes were then incubated with secondary antibodies for 1 h at RT. The signals were detected using ECL-Plus Western blot detection system.

### Acridine Orange and LysoTracker Deep Red Staining

Cells grown on coverslips were incubated with 5 μg/ml acridine orange or 100 nM LysoTracker Deep Red at 37°C for 30 min after infected with *S. aureus* for 2, 4, 6, and 8 h. The fluorescence signal of acridine orange and LysoTracker Deep Red was subsequently observed under a confocal microscope.

### Transmission Electron Microscopy

After fixing with 2.5% glutaraldehyde and 5% formaldehyde for at least 2 h, the samples were washed thrice with 0.1 M phosphate buffer solution. The samples were fixed with 1% osmium tetroxide for 2 h. The above operations were all performed at 4°C. Following, dehydration was performed in stages in 50, 70, 80, 90, and 100% acetone for 15 min. The embedding solution and propylene oxide were 1:1 at normal temperature for 1 h, and the embedding solution and propylene oxide were 3:1 at normal temperature for 5 h. The embedding solution was saturated on a shaker for 5 h at normal temperature. Finally, it was left to stand at 37°C for 12 h and transferred to 45°C for 24 h, then to 60°C for 24 h for curing. Ultrathin sections were then prepared and stained. Samples were examined in a Zeiss TEM 910 (Zeiss, Oberkochen, Germany) at an acceleration voltage of 80 kV and at calibrated magnifications. Images were recorded digitally at calibrated magnifications with a slow-scan charge-coupled device (CCD) camera (ProScan, 1,024 × 1,024, Scheuring, Germany) with ITEM Software (Olympus Soft Imaging Solutions, Münster, Germany).

## Results

### Cell Infection Model Successfully Constructed

**FIGURE 1:**
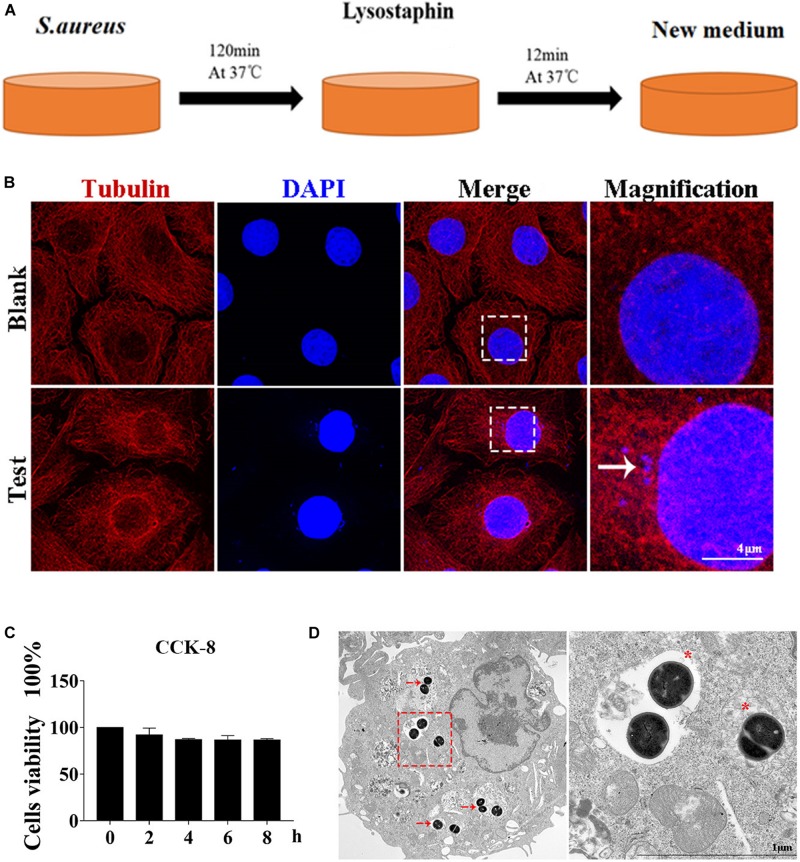
Cell infection model successfully constructed. **(A)** Schematic of experimental design. Eukaryotic cells and *S. aureus* were incubated at 37°C for 120 min. Lysostaphin was then added to kill all bacteria outside the cell. After incubation at 37°C for 12 min, the culture medium was changed. **(B)** Representative confocal images showing colocalization of tubulin with 4’,6-diamidine-2’-phenylindole dihydrochloride (DAPI). High magnification images of the outlined area are shown on the right. “→” points to *S. aureus*. Scale bars: 4 μm. **(C)** Effect of *S. aureus* invasion time on cell activity in MAC-T cells. Values were shown as means ± SD, *n* = 3. **(D)** MAC-T cells were infected with *S. aureus*, fixed 2 hpi, and analyzed by transmission electron microscopy. “→” points to *S. aureus*. “*” is a double-layer membrane. Scale bars: 200 nm.

Autophagy caused by intracellular *S. aureus* in MAC-T was explored. Methods in a previous study (10) were slightly modified to establish an intracellular infection model. Incubation at 37°C for 2 h can effectively enable *S. aureus* invasion of MAC-T cells, and lysostaphin (100 μg/ml) can effectively kill the extracellular *S. aureus*. The cell infection model was successfully constructed after 12 min of lysostaphin treatment (Figure 1A).

As shown in Figure 1B, *S. aureus* was observed in the MAC-T of the test group but not in the blank control group. In addition, CCK-8 assay indicated that continuous infection with *S. aureus* for 8 h did not affect cell activity (Figure 1C). Through transmission electron microscope analysis, the *S. aureus* bacteria in MAC-T were presented along with the autophagic vesicle membrane structure around the bacteria (Figure 1D).

### Intracellular *S. aureus* Induced Autophagy

**FIGURE 2:**
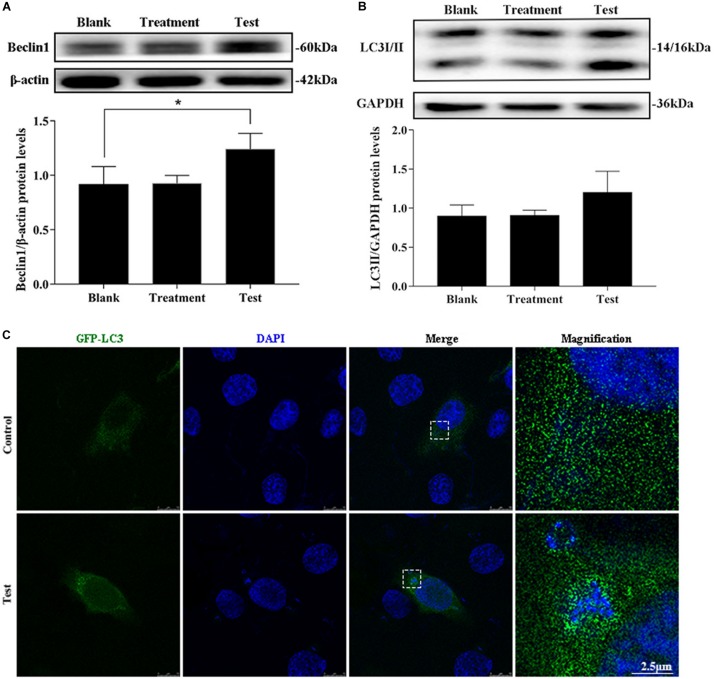
Intracellular *S. aureus* induced the occurrence of autophagy. **(A)** Protein level of Beclin1 in MAC-T cells with different treatments. **(B)** Protein level of light chain 3II (LC3II) in MAC-T cells with different treatments. Upper panels: representative Western blot images; lower panels: quantitative analysis (mean ± SEM, *n* = 3, *p < 0.05). **(C)** Formation of GFP-LC3 puncta was observed under the confocal microscope. Scale bars: 2.5 μm.

The treatment group was the cells treated with lysostaphin alone, and autophagy did not occur in these cells. However, infection with *S. aureus* resulted in a substantial increase in Beclin1 expression (Figures 2A,B). The conversion of 1 light chain 3I LC3I to LC3II also increased (*p* = 0.637). Intuitively, the green fluorescent spots of green fluorescent protein (GFP)-LC3 clustered around the intracellular bacteria in the test group, whereas the blank group showed a diffuse distribution (Figure 2C). This outcome suggested that *S. aureus* induce autophagy in MAC-T.

### Autophagy Flux Was Blocked

**FIGURE 3:**
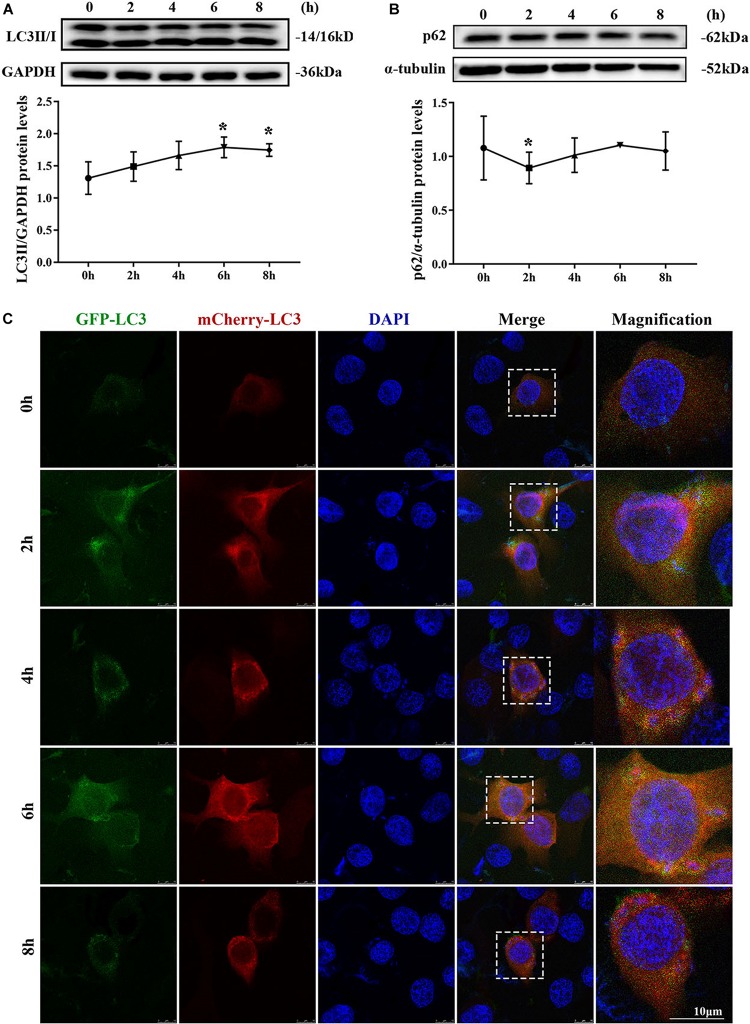
Autophagy flux was blocked. **(A)** Light chain 3II (LC3II) protein level in MAC-T cells. **(B)** SQSTM1/p62 protein level in MAC-T cells. Upper panels: representative Western blot images; lower panels: quantitative analysis (mean ± SEM, *n* = 3, *p < 0.05). Cells grown on coverslips were transfected with mCherry-GFP-LC3 for 36 h, then infected with *S. aureus* at different times to monitor the autophagic flux. Representative confocal images were presented in **(C)**. Scale bars: 10 μm.

MAC-T cells were continuously infected for 8 h to observe the occurrence of dynamic autophagy. The ratio of LC3II/GAPDH showed an increasing trend by Western blotting, and the protein expression level of SQSTM1/p62 presented a similar trend after 4 h of infection (Figures 3A,B). The results of the cells transfected with mCherry-GFP-LC3 indicated that yellow mottled fluorescence tended to increase within 6 h as the infection time extended, whereas red mottled fluorescence became excessed and peaked at the eighth hour (Figure 3C). This observation suggested that autophagy flux was blocked during *S. aureus* infection.

### *S. aureus* Causes Increased pH in Lysosomes

**FIGURE 4:**
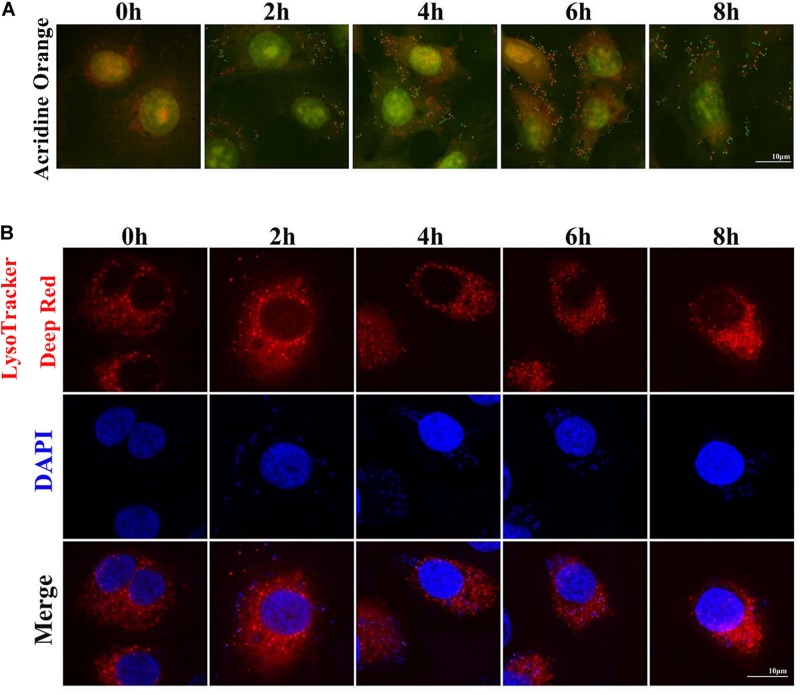
*Staphylococcus aureus* causes increased pH in lysosomes. **(A)** Cells stained with 100 nM LysoTracker Deep Red at 37°C for 30 min after *S. aureus* infection to assess the lysosomal pH. **(B)** Cells stained with 10 μg/ml acridine orange at 37°C for 30 min. Slides were viewed using a scanning confocal microscope, and representative confocal images were shown. Scale bars: 10 μm.

Two sensitive lysosome pH probes were used to monitor lysosome pH changes in MAC-T (Figure 4). First, acridine orange staining showed that cytoplasm presented diffuse fluorescence with green color. Lysosomes showed red fluorescence, and intracellular live bacteria reflected green mottled fluorescence. The intracellular red fluorescence was enhanced during the second to sixth hour postinfection. LysoTracker Deep Red displayed red fluorescence in lysosomes in a pH-dependent manner, and fluorescence enhancement indicates a decrease in lysosome pH. Similarly, red fluorescence around intracellular *S. aureus* was reduced at the fourth and sixth hour postinfection. These results indicated that infection with *S. aureus* increased the pH in lysosomes.

### Degradation of Lysosomes Was Impaired

**FIGURE 5:**
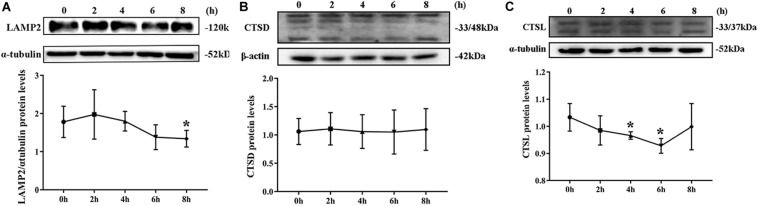
Degradation of lysosomes was impaired. **(A)** Protein level of lysosomal-associated membrane protein 2 (LAMP2) in MAC-T cells treated at different times. **(B)** Cathepsin D (CTSD) expression level in MAC-T cells treated at different times. **(C)** Cathepsin L (CTSL) expression level in MAC-T cells treated at different times. Upper panels: representative Western blot images; lower panels: quantitative analysis (mean ± SEM, *n* = 3, *p < 0.05).

By Western blotting, LAMP2 protein level substantially decreased from the second hour postinfection (Figure 5A). By contrast, no remarkable change was noted in CTSD protein level during *S. aureus* infection (Figure 5B). The expression of CTDL protein also decreased in the beginning of infection but recovered at the eighth hour (Figure 5C). These outcomes confirmed that lysosomal degradation was impaired after *S. aureus* infection of MAC-T.

## Discussion

*Staphylococcus aureus* mastitis is caused mainly by the pathogens entering the lobules through the milk ducts (23). First, *S. aureus* adheres to the mammary epithelial cells by adhesion molecules, then settles down and multiplies (24). *S. aureus* can invade cells to evade the body’s immune defense and survive in the cells (25). As the “cell guard,” autophagy plays a role in removing foreign pathogens (9). Moreover, the integrity of autophagy affects the cells’ defense against pathogens. Therefore, exploring the interaction between *S. aureus* and autophagy flux in BMECs is important.

Autophagosome formation is the first step of autophagy flux. In an attempt to visualize *S. aureus* inside the classical vesicle of autophagosome compartments, transmission electron microscopy was used. *S. aureus* cells residing within a double membrane structure were not identified, but other types of *S. aureus* containing vesicles were observed as well as *S. aureus* residing freely in the cytoplasm (Figure 1D). *S. aureus* were undergoing replication in cell centrally placed inside a multivesicular body and enclosed inside a single membrane compartment. Another type of intracellular compartment containing *S. aureus* was observed. A spacious vesicle not only held a bacterial cell but also contained cytoplasmic (membranous) material that might have resulted from intraluminal vesicle formation or from fusion with autophagic vesicles. The structure originated from phagocytosis and not from xenophagy. Finally, replicating *S. aureus* cells were spotted inside vesicles as well as within the cytoplasm. These findings are consistent with the results of previous studies on *Salmonella typhimurium* (26). These results identified the occurrence of cellular autophagy and bacterial escape.

Beclin1, which functions as a molecular scaffold that binds with other Atg regulatory proteins, regulates autophagy levels and locates autophagosomes (27). Beclin1 expression enhancement can be used as an important index to evaluate the increase in autophagy level. The results showed that the expression of Beclin1 protein substantially increased after infection with *S. aureus*. LC3 is the most widely used molecular marker of autophagosome in current studies because it can specifically locate the autophagosome membrane. Furthermore, this study found that the amount of LC3II was related to the intensity of autophagy (28). GFP-LC3 transfected cells are a common tool for autophagy evaluation. In this study, LC3II expression level increased after *S. aureus* invaded MAC-T cells. These results suggested that *S. aureus* could induce autophagy in BMECs.

The fusion of autophagosomes and lysosomes is critical in autophagic flux. SQSTM1/p62 is the most critical “truck protein” for selective autophagy, and they are also known as selective autophagy receptors (29). SQSTM1/p62 acts as a linker protein to mediate the degradation of its recognition substrate (30). The enhanced SQSTM1/p62 protein level is considered a symbol that the autophagy flux is blocked (28). A previous study showed that the fusion of autophagosomes and lysosomes was hindered by *S. aureus* and that the formation of autophagosomes was conducive to the intracellular survival of bacteria (31). The results of the present study revealed that the protein expression level of SQSTM1/p62 began to rise from the second hour during continuous infection of *S. aureus* in MAC-T. The mCherry-GFP-LC3 is an adenovirus specifically designed to detect the levels of autophagic flux (32). The present study observed that mCherry-GFP-LC3 showed yellow fluorescence accumulation after bacterial infection. From the above results, *S. aureus* infection blocks the autophagy flux in BMECs by interfering with the fusion of autophagosomes and lysosomes.

Lysosomal degradation also affects autophagy flux. Under normal physiological conditions, lysosomes are weakly acidic. Considering the acidic environment of lysosomes, a large number of proteolytic enzymes can play a role in degradation (33). LysoTracker Deep Red is a fluorescent probe that can accumulate in acidic vesicles. The intensity and quantity of red fluorescence represent the pH and quantity of lysosomes (34, 35). In the current study, the red fluorescence weakened, and the number of spots decreased at the fourth and sixth hour of *S. aureus* infection. Acridine orange can stain the double-stranded DNA green and single-stranded RNA red. In live cells, acridine orange can be accumulated by acidic vesicles that yield prominent red signals (35). Our results showed that the red fluorescence decreased sharply at the eighth hour after *S. aureus* infection, suggesting that once *S. aureus* infected MAC-T cells, the pH in lysosome increased. LAMP2 is the main protein on the lysosome membrane. LAMP2 not only plays an important role in protecting the integrity of the lysosome membrane but also participates in regulating the fusion of autophagy vesicles and lysosomes. When autolysosome pathway was activated, the LAMP2 expression level increased and located on the perinuclear lysosome (20). In this study, Western blot results showed that the LAMP2 protein expression level decreased gradually after *S. aureus* infection. CTSD is an aspartic acid-like lysosomal peptide endonuclease whose normal function is to hydrolyze hormones, peptide precursors, peptides, and structural and functional proteins in the acidic environment of lysosomes (36, 37). CTSL is the main member of the lysosomal cysteine protease family. CTSL can hydrolyze proteins, plasma proteins, hormones, and phagocytic bacteria by activating CTSB (38). The present study found that *S. aureus* infection of MAC-T did not affect the expression of CTSD but inhibited that of CTSL. This finding indicated that the invasion of *S. aureus* led to impaired degradation of lysosomes.

## Data Availability Statement

The datasets generated for this study are available on request to the corresponding author.

## Author Contributions

YL: conceptualization. NG: data curation, investigation, and writing—original draft. XW and XY: formal analysis. NG and RW: methodology. JL and YL: project administration, writing, reviewing, and editing. YZ: software. JL: supervision. YL and MZ: validation. XW: visualization.

## Conflict of Interest

The authors declare that the research was conducted in the absence of any commercial or financial relationships that could be construed as a potential conflict of interest.
